# Chondroprotective Effects of Ginsenoside Rg1 in Human Osteoarthritis Chondrocytes and a Rat Model of Anterior Cruciate Ligament Transection

**DOI:** 10.3390/nu9030263

**Published:** 2017-03-10

**Authors:** Wendan Cheng, Juehua Jing, Zhen Wang, Dongying Wu, Yumin Huang

**Affiliations:** 1 Department of Orthopedics, The Second Hospital of Anhui Medical University, No. 678 Furong Road, Hefei 230601, China; sunyccc@126.com (W.C.); jjhhu@sina.com (J.J.); 2 Department of Orthopedics, Lu’an People’s Hospital Affiliated to Anhui Medical University, Lu’an 237000, China; 3 Department of Orthopedics, The Peoples Hospital of Luhe Affiliated to Yangzhou University Medical Academy, Nanjing 211500, China; dzwangzhen@126.com; 4 Department of Orthopedics, The Affiliated Hospital of Xuzhou Medical University, No. 99 Huaihai West Road, Xuzhou 221000, China; 5 Department of Orthopedics, The First Affiliated Hospital of Nanjing Medical University, No. 300 Guangzhou Road, Nanjing 210029, China

**Keywords:** ginsenoside-Rg1, osteoarthritis, chondrocyte

## Abstract

This study aimed to assess whether Ginsenoside Rg1 (Rg1) inhibits inflammatory responses in human chondrocytes and reduces articular cartilage damage in a rat model of osteoarthritis (OA). Gene expression and protein levels of type II collagen, aggrecan, matrix metalloproteinase (MMP)-13 and cyclooxygenase-2 (COX-2) were determined in vitro by quantitative real-time-polymerase chain reaction and Western blotting. Prostaglandin E2 (PGE_2_) amounts in the culture medium were determined by enzyme-linked immunosorbent assay (ELISA). For in vivo assessment, a rat model of OA was generated by anterior cruciate ligament transection (ACLT). Four weeks after ACLT, Rg1 (30 or 60 mg/kg) or saline was administered by gavage once a day for eight consecutive weeks. Joint damage was analyzed by histology and immunohistochemistry. Ginsenoside Rg1 inhibited Interleukin (IL)-1β-induced chondrocyte gene and protein expressions of MMP-13, COX-2 and PGE_2_, and prevented type II collagen and aggrecan degradation, in a dose-dependent manner. Administration of Ginsenoside Rg1 to OA rats attenuated cartilage degeneration, and reduced type II collagen loss and MMP-13 levels. These findings demonstrated that Ginsenoside Rg1 can inhibit inflammatory responses in human chondrocytes in vitro and reduce articular cartilage damage in vivo, confirming the potential therapeutic value of Ginsenoside Rg1 in OA.

## 1. Introduction

Osteoarthritis (OA) is a pathological process characterized by degenerative changes and inflammatory responses in chondrocytes. Interleukin (IL)-1β, a major pro-inflammatory cytokine produced by chondrocytes and synovial cells, contributes to increased chondrocyte apoptosis [1,2,3], along with the synthesis of other inflammatory mediators, including matrix metalloproteinase (MMP), cyclooxygenase-2 (COX-2) and prostaglandin E2 (PGE_2_). These inflammatory mediators ultimately inhibit type II collagen and aggrecan synthesis, increase extracellular matrix (ECM) degradation, and cause articular cartilage damage [4,5,6,7]. According to the 2015 recommendations for knee OA, the primary pharmacotherapy for OA currently comprises non-steroidal anti-inflammatory drugs (NSAIDs) and hormone-like drugs. However, these drugs are only temporarily effective and exhibit numerous side effects. Therefore, safe, effective and economical strategies to inhibit both apoptosis and inflammation in chondrocytes for the treatment of OA are urgently required.

Ginseng is broadly used as an herbal medicine because of its wide-range efficacy and low side-effect profile [8,9]. Among the 30 ginsenosides, ginsenoside Rg1 (Rg1) is one of the major active ingredients of ginseng [10]. Rg1 is a proven effective agent for neurodegenerative diseases such as Alzheimer’s disease, and exerts remarkable neuroprotective effects against tert-Butylhydroperoxide induced oxidative stress [11,12]. In addition, our previous study demonstrated that Rg1 protects chondrocytes from IL-1β-induced apoptosis via the phosphatidylinositol 3-kinase/protein kinase B signaling pathway, by preventing caspase-3 release [13]. These findings suggest that Rg1 may serve as a novel pharmacotherapeutic drug for OA. However, in addition to chondrocyte apoptosis, inflammation in chondrocytes plays a critical role in the pathogenesis of OA. The anti- inflammatory properties in chondrocytes and protective effects on OA in vivo of Rg1 have not been reported.

The present study assessed the ability of Rg1 to inhibit inflammatory responses and reduce articular cartilage damage in OA by using an in vitro model of human chondrocytes and an in vivo model of rat OA, and confirmed the potential therapeutic value of Rg1 in OA. 

## 2. Materials and Methods

### 2.1. Materials

Ginsenoside Rg1 (>98% purity) was purchased from the National Institutes for Food and Drug Control (Beijing, China). Recombinant human IL-1β was from R&D Systems (Minneapolis, MN, USA). Type II collagen and MMP-13 antibodies used in immunohistochemistry were obtained from Boster (Wuhan, Hubei, China) and Abcam (Cambridge, MA, USA), respectively. All other antibodies were purchased from Santa Cruz Biotechnology Inc. (Santa Cruz, CA, USA). Enzyme-linked immunosorbent assay (ELISA) kits were from Enzo life sciences (Farmingdale, NY, USA). 3-(4,5-dimethylthiazal-2-yl)-2,5-diphenyl-tetrazolium bromide (MTT) was obtained from Sigma (St. Louis, MO, USA).

### 2.2. Cell Culture

The study protocol was approved by the Ethics committee of The First Affiliated Hospital to Nanjing Medical University; all patients involved provided the necessary informed consent for participation and publication (permit number 20120413). Articular chondrocytes were harvested from 20 patients who underwent total knee replacement. NSAIDs were dissolved at least 1 week prior to surgery. Isolated chondrocytes were cultured in Dulbecco’s modified Eagle medium (DMEM) supplemented with 10% fetal bovine serum (FBS) at 37 °C in a 5% CO_2_ incubator. First-generation cells were cultured in tissue culture dishes at a density of 1 × 10^6^ cells/mL.

### 2.3. Animals

Forty-eight male Sprague-Dawley (SD) rats (Animal Inc., Nanjing Medical University, Nanjing, Jiangsu, China) weighing 240–280 g were housed in a controlled-temperature room (21–22 °C) (six per cage), with free access to food and water. To confirm the therapeutic potential of Rg1 in an OA model, forty-eight rats were randomized into four groups of 12 rats each. All animal procedures were approved by the Animal Research Ethics Committee of Nanjing Medical University.

### 2.4. Anterior Cruciate Ligament Transaction (ACLT)

The ACLT model was established as previously described [14]. Briefly, rats were anesthetized with chloral hydrate. The right knee joint was exposed through a medial parapatellar approach. The anterior cruciate ligament (ACL) was then transected with micro-scissors. Complete transection was confirmed by a positive anterior drawer sign. The control group underwent arthrotomy without transection of the ACL. After surgery, all animals were allowed free exercise out of their cages for 20 min daily.

### 2.5. Experimental Design

For the in vitro study, first-generation human chondrocytes were cultured in serum-free DMEM supplemented with 2% serum-free bovine serum albumin (BSA) for 12 h. To assess the anti-inflammatory effects of different concentrations of Rg1, cells were treated with 10 ng/mL IL-1β, alone or in combination with Rg1 at 0.1, 1 and 10 μg/mL. A control group was left untreated except for medium change. Cells were harvested after 24 h of incubation.

For in vivo assessment, rats underwent anterior cruciate ligament transaction as described above. Four weeks after ACLT, Rg1 intervention groups (R1 group, 30 mg/kg/day; R2 group, 60 mg/kg/day) received Rg1 dissolved in sterile saline by gavage once a day for eight consecutive weeks. Meanwhile, rats in control and ACLT groups were administered an equivalent volume of sterile saline. All animals were sacrificed 12 weeks post-surgery, and right knees were dissected to observe articular cartilage changes and for histological and immunohistochemical analyses.

### 2.6. Quantitative Real-Time-Polymerase Chain Reaction (q-PCR)

Total RNA was extracted with RNAiso Plus (TaKaRa, Dalian, China)), and reverse transcribed into cDNA using the PrimeScript RT reagent kit (TaKaRa, Dalian, China). Quantitative real-time PCR analysis was performed with SYBR Premix Ex Taq II reagents from TaKaRa (Dalian, China), according to the manufacturer’s instructions. PCR reactions were performed in a 20 μL mixtures containing 2 μL of cDNA. Aggrecan, type II collagen, MMP-13, COX-2, PGE_2_ and β-actin cDNAs were amplified using specific primers (TaKaRa). Gene expression was normalized to glyceraldehyde-3-phosphatedehydrogenase (GADPH) and derived by the 2^-∆∆CT^ method.

### 2.7. Western Blotting

Total protein was prepared using RIPA buffer (Beyotime, Haimen, Jiangsu, China); nuclear protein isolation was carried out with Nuclear Protein Extraction Kit (Beyotime) according to the manufacturer’s instructions. Protein mixtures were separated by sodium dodecyl sulfate-polyacrylamide gel electrophoresis and transferred onto nitrocellulose membranes. The membranes were incubated with antibodies raised against the proteins of interest and detected by enhanced chemiluminescence (Thermo Fisher Scientific, Rockford, IL, USA). The data were analyzed with the Image Lab software (Bio-Rad Laboratories, Hercules, CA, USA). 

### 2.8. ELISA

An ELISA kit (Enzo Life Sciences, Farmingdale, NY, USA) was used to measure PGE_2_ levels in the culture medium, according to the manufacturer’s protocol. 

### 2.9. Histology and Immunohistochemistry

The rats in each group were sacrificed by cervical dislocation under anesthesia at 12 weeks after the surgery. The patella was isolated to expose the knee joint cavity. The fixed specimens were decalcified using 10% ethylenediaminetetraacetic for 18 days. After decalcification, the specimens were paraffin embedded. Standardized 3 μm serial sections were cut in the sagittal plane and stained with hematoxylin and eosin (H&E), Safranin O/fast green, and toluidine blue.

To analyze type II collagen and MMP-13 distribution in the cartilage, the sections were de-paraffinized and rehydrated in graded ethanol. After incubation with antibodies targeting rat type II collagen and MMP-13, the samples were treated with secondary antibodies. Finally, the specimens were photographed under a microscope (Olympus, Tokyo, Japan). Percentages of MMP-13-positive cells in cartilage samples were obtained in six fields at 200× magnification. Cell counts were performed by three independent observers, and mean values were used as the final scores.

A modified Mankin histological score was used to assess histological injuries of the articular cartilage as follows. Structural changes were scored on a scale of 0–6: 0 normal; 1 irregular surface, including fissures into the radial layer; 2 pannus; 3 absence of superficial cartilage layers; 4 slight disorganization (absent cell row and small superficial clusters); 5 fissure into the calcified cartilage layer; 6 disorganization (chaotic structure, clusters, and osteoclast activity). Cellular abnormalities were scored on a scale of 0–3: 0 normal; 1 hypercellularity, including small superficial clusters; 2 clusters; 3 hypocellularity. Matrix staining was scored on a scale of 0–4: 0 normal/slight reduction in staining; 1 reduced staining in the radial layer; 2 reduced staining in the interterritorial matrix; 3 staining present only in the pericellular matrix; 4 no staining [15]. Three independent observers assessed cartilage damage in a blinded manner.

### 2.10. Statistical Analysis

Data are mean ± Standard Error of Mean (SEM) unless otherwise stated. Gene expression, Western blot, MTT assay and immunohistochemistry data were analyzed by one-way Analysis of Variance followed by Dunnett’s multiple comparisons. Differences in Mankin scores were assessed by the Kruskal–Wallis test. Dunnett’s multiple comparison test was used to assess MMP-13-positive chondrocytes. *p* < 0.05 was considered statistically significant. Statistical analyses were performed with the SPSS software version 16.0 (SPSS Inc., Chicago, IL, USA) or GraphPad Prism (GraphPad Software, San Diego, CA, USA).

## 3. Results

### 3.1. Effects of Rg1 on Gene Expression of Extracellular Matrix and Inflammatory Mediators after Induction by IL-1β

**Figure 1:**
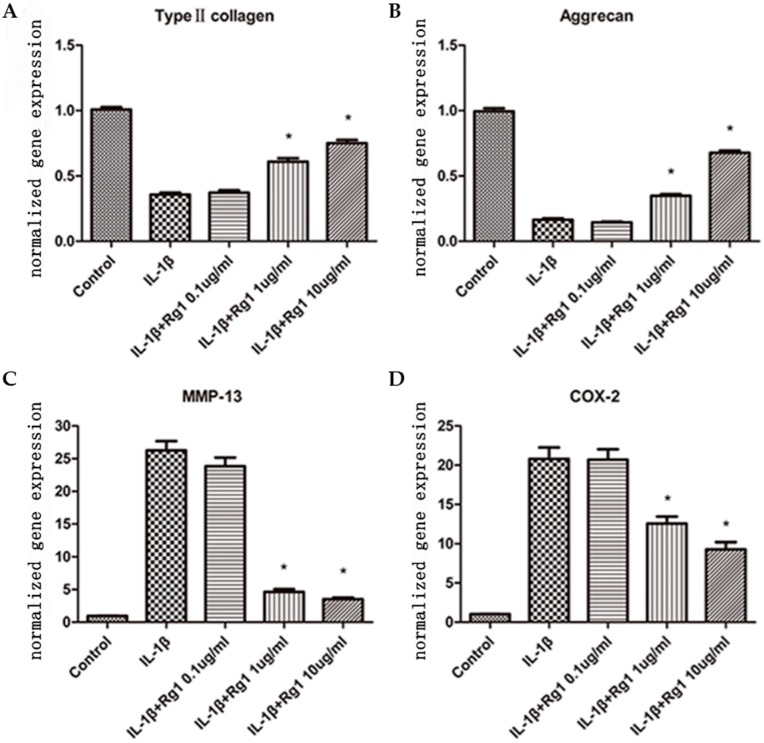
Effect of Ginsenoside Rg1 (Rg1) on gene expression levels of extracellular matrix and inflammatory mediators after induction by Interleukin (IL)-1β. Human osteoarthritis (OA) chondrocytes were treated with the medium (control group), and IL-1β (10 ng/mL) alone or in combination with Rg1 (0.1, 1, or 10 μg/mL). Gene expression levels of type II collagen (**A**), aggrecan (**B**), matrix metalloproteinase (MMP)-13 (**C**) and cyclooxygenase-2 (COX-2) (**D**) were determined by quantitative real-time PCR, normalized to glyceraldehyde-3-phosphatedehydrogenase (GADPH) and expressed as means ± Standard Error of Mean (SEM) of four independent experiments. * *p* < 0.05 compared with cells treated with IL-1β alone.

Gene expression levels of type II collagen (Figure 1A) and aggrecan (Figure 1B) in the IL-1β group were reduced after treatment. They were increased by 1.7- and 2.1-fold after treatment with 1 μg/mL Rg1, and by 2.1- and 4.1-fold after treatment with 10 μg/mL Rg1. MMP-13 (Figure 1C) and COX-2 (Figure 1D) mRNA amounts in the IL-1β group were increased; they were reduced by 5.6- and 1.6-fold, and 7.5- and 2.2-fold, respectively, after treatment with 1 μg/mL and 10 μg/mL Rg1 (all *p* < 0.05). However, no effect was observed at 0.1 μg/mL Rg1. Thus, Rg1 effects were dose-dependent.

### 3.2. Effects of Rg1 on Protein Expression of Extracellular Matrix and Inflammatory Mediators after Induction by IL-1β

**Figure 2:**
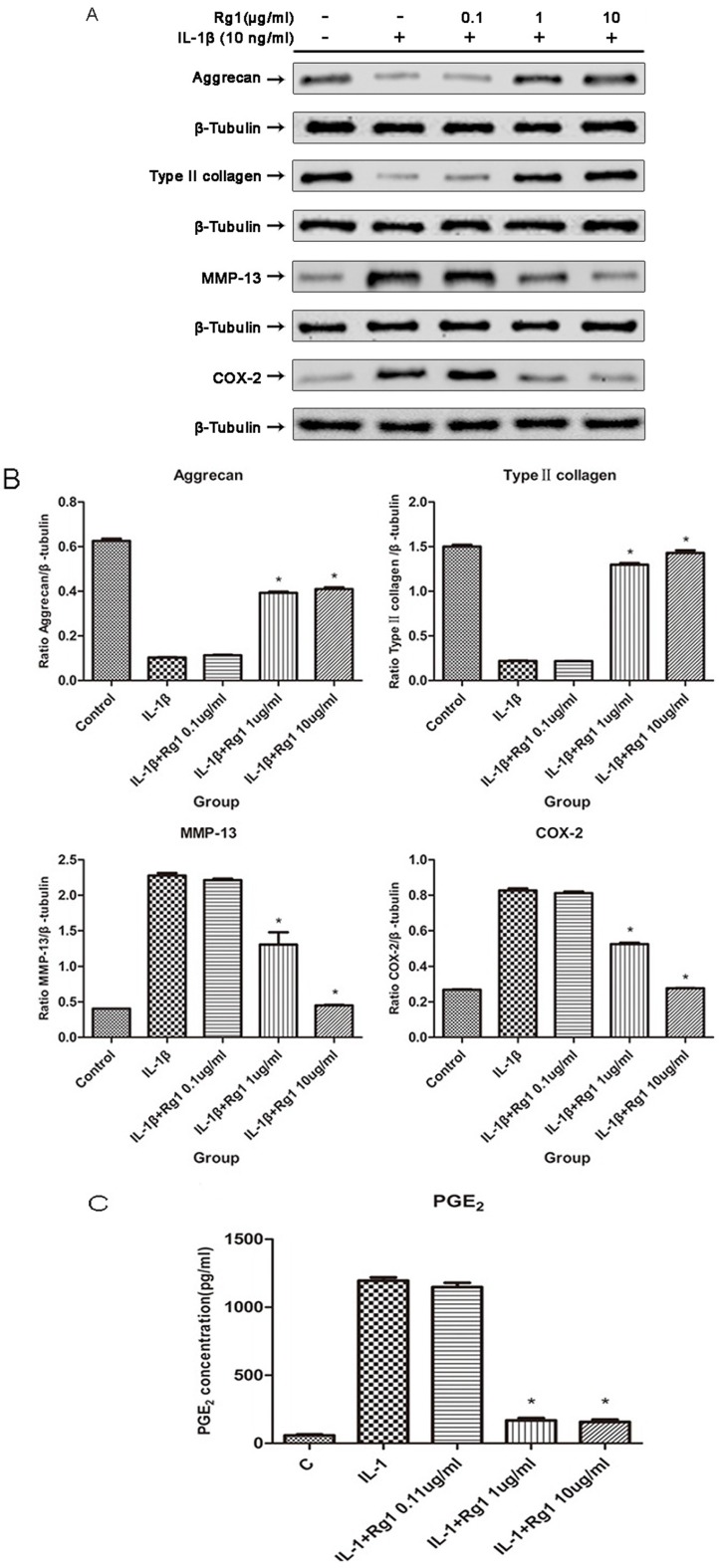
Effect of Rg1 on protein levels of extracellular matrix and inflammatory mediators after induction by IL-1β. Human OA chondrocytes were treated with medium (control group), and IL-1β (10 ng/mL) alone or in combination with Rg1 (0.1, 1, or 10 μg/mL). Total protein was extracted for Western blot analysis. The following proteins were assessed: type II collagen, aggrecan, MMP-13, COX-2 and β-Tubulin (**A**); the relative protein levels of type II collagen, aggrecan, MMP-13 and COX-2 were quantified by densitometric analysis and normalized to β-tubulin (**B**); Prostaglandin E2 (PGE_2_) concentrations in the corresponding culture media were measured by enzyme-linked immunosorbent assay (ELISA) (**C**). Data are mean ± SEM of four independent experiments. * *p* < 0.05 compared with cells treated with IL-1β alone.

Protein levels of type II collagen, aggrecan, MMP-13, and COX-2 were analyzed by Western blotting (Figure 2A), and quantified by densitometry (Figure 2B). Protein levels of both type II collagen and aggrecan were reduced by IL-1β treatment; administration of 1 or 10 μg/mL Rg1 resulted in increased amounts of these proteins. Analysis of MMP-13 and COX-2 by Western blotting, alongside PGE_2_ amount assessment by ELISA (Figure 2C), revealed that the levels of all three proteins increased significantly in the IL-1β-treatment group, and significantly inhibited by Rg1 at 1 or 10 μg/mL.

### 3.3. Rat OA and Gross Morphology

**Figure 3:**
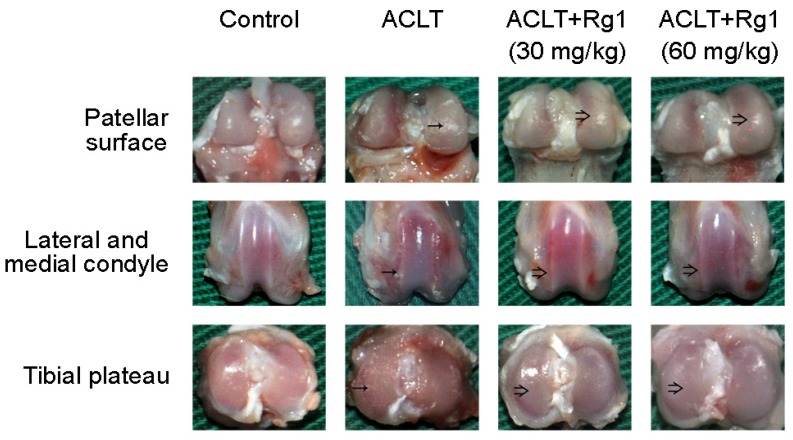
Rat OA and gross morphology. OA was induced by Anterior Cruciate Ligament Transaction (ACLT) of the right knee. Four weeks after ACLT, Rg1 intervention groups received Rg1 (30 or 60 mg/kg/day) by gavage once daily for eight consecutive weeks. Gross morphological changes of femoral condyles and the tibial plateau were photographed to compare cartilage lesions at 12 weeks after surgery. “→” indicates articular surface abrasion; “⇒” indicates articular cartilage repair.

Visible abrasion of the articular surface was detected in the right knee joint of OA rats (Figure 3). Compared with the ACLT group, cartilage destruction was slightly improved in the R1 group, with partial repair on the articular surface. However, slight cartilage erosion was still detected on the tibial plateau, the lateral and medial condyles, and patellar surface (Figure 3). The situation was further improved in the R2 group, which showed smoother and more regular articular surface (Figure 3). 

### 3.4. Histology and Immunohistochemistry Findings

**Figure 4:**
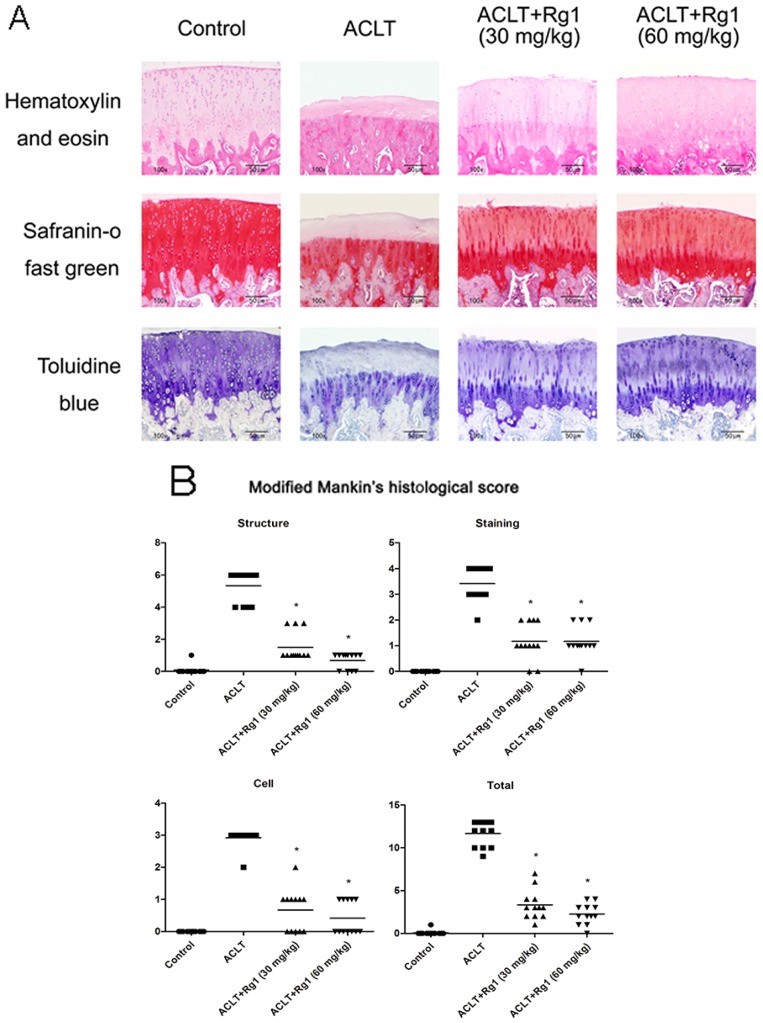

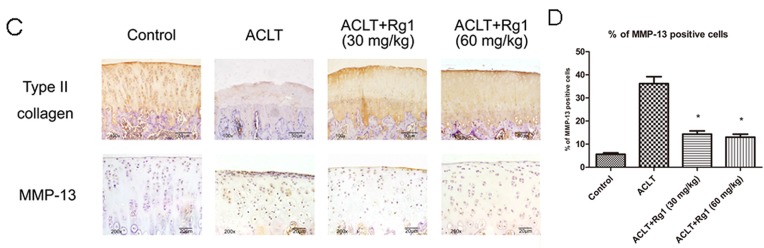
Histological and immunohistochemical findings. Four weeks after ACLT, Rg1 intervention groups received Rg1 (30 or 60 mg/kg/day) by gavage once daily for eight consecutive weeks. (**A**) representative photomicrographs of hematoxylin and eosin (H&E), Safranin O-fast green, and toluidine blue -stained tissue sections of knee joint specimens from OA rats treated with Rg1 or saline (original magnification ×100); (**B**) joint lesions were graded on a scale of 0–13 using the modified Mankin scoring. They were graphed as dot plots with the mean (bar) of 12 rats per group; (**C**) representative photomicrographs of immunohistochemical staining for type II collagen (original magnification ×100) and MMP-13 (original magnification ×200). Yellow staining indicates collagen type II expression in the cartilage. MMP-13 positive cells are stained brown; and (**D**) bar graphs represent mean percentage ± SEM of MMP-13-positive cells in 12 rats per group. * *p* < 0.05 compared with the ACLT group.

Structural changes in the joints as well as aggrecan content were evaluated histologically (Figure 4A); type II collagen and MMP-13 (Figure 4C) levels were analyzed by immunohistochemistry. The ACLT group showed serious cartilage destruction, irregular abrasions of the cartilage surfaces, chondrocyte disappearance, and aggrecan depletion. Type II collagen showed similar changes as aggrecan, as demonstrated by immunohistochemistry. No Safranin O/fast green or toluidine blue staining was evident, and remarkably more MMP-13-positive chondrocytes (Figure 4D) were found in the OA cartilage specimens. These histo-morphological and immunohistochemical changes were reduced in the R1 group and further decreased in the R2 group. The modified Mankin scores (Figure 4B) were significantly higher in the ACLT group compared with the control values, and lower in the R1 and R2 groups than the ACLT group. Percentages of MMP-13-positive cells were higher in the ACLT group than the control, R1 and R2 groups.

## 4. Discussion

Our previous study demonstrated that Rg1 protects chondrocytes from IL-1β-induced apoptosis. However, inflammatory responses induced by IL-1β in chondrocytes also play a critical role in the initiation and development of OA [7]. The anti-inflammatory effects of Rg1 on chondrocytes and protective effects in osteoarthritis had not been reported. 

Hitherto, more than 30 ginsenosides have been identified from ginseng [16]. Ginsenosides are classified into 20 (*S*)-protopanaxadiol and 20 (*S*)-protopanaxatriol groups. Rg1 is one of the most abundant ginsenosides in ginseng and categorized into the 20 (*S*)-protopanaxatriol group [17]. After systematic review, no published article was found on the human use of the bioavailable Rg1 monomer. Several studies focused on its bioavailability in rats in vivo; in vitro studies demonstrated that Rg1 can be absorbed after oral gavage [18]. A preliminary study showed that ginsenoside Rg1 is metabolized into 20 (*S*)-protopanaxatriol via ginsenoside Rh1 and ginsenoside F1 by the gut microbiota of humans and mice. These metabolites may ameliorate inflammatory diseases, such as colitis, by inhibiting the binding of lipopolysaccharide to toll-like receptor 4 on peritoneal macrophages and restoring the Th17/Treg cell balances [19].

MMP-13 plays a pivotal role in initiating the degradation of type II collagen [20]. Previous studies have shown that herbs can decrease MMP-13 expression, when administered as complementary OA treatment [7,21]. Senescent chondrocytes have been detected in damaged OA cartilage samples [22]. The current view is that the local balance of MMP and tissue inhibitor of metalloproteinase activities is pivotal in determining the extent of ECM turnover. Thus, a hypothesis was formed that chondrocytes in aging or diseased cartilage may become senescent, with associated phenotypic changes contributing towards the development or progression of OA [22]. While serving as pro-senescent compounds in cancer cell models [23,24], ginsenoside Rg1 was reported to exhibit protective effects against IL-1β, H_2_O_2_, and tert-Butyl hydroperoxide-induced senescence in endothelial progenitor cells [25] and fibroblasts [26]. In the current study, Rg1 decreased IL-1β-induced MMP-13 expression. This inhibition may be crucial for inhibiting ECM degradation. The impact of ginsenoside Rg1 on modulating chondrocyte senescence need to be further explored.

PGE_2_ can influence chondrocyte metabolism, degrade type II collagen, and cause articular cartilage degeneration. Thus, it can be considered a putative therapeutic target for OA [27]. As shown above, Rg1 inhibited COX-2 and PGE_2_ protein levels, in a similar manner as symptomatic slow-acting drugs of OA, which also ameliorated OA symptoms by inhibiting the expression of COX-2 and PGE_2_ [28,29]. We predict that these effects of Rg1 could be crucial in slowing OA progression.

The rat model of ACLT has been used previously to assess tissue alterations in OA [30,31,32]. Reported findings demonstrate similarities between ACLT induced OA in rats and human OA, including type II collagen and aggrecan degradation, and chondrocyte loss, leading to progressive articular cartilage degeneration [33,34]. We found that Rg1 protected the cartilage and ECM. In addition, Rg1 inhibited OA progression association with reduced MMP-13 expression in the OA model. This phenomenon may represent one of the mechanisms, whereby Rg1 mitigates cartilage and ECM damage.

Rg1 is composed of a steroidal skeleton with sugar moieties, as demonstrated by Chan et al., and exhibits estrogen-like effects [35,36,37]. In addition to bone-inducing effects, estrogen sustains normal chondrocyte metabolism and reduces the sensitization of articular cartilage to OA pathogenic factors [20]. Thus, we suggest that Rg1 may exert estrogen-like effects in OA treatment.

Rg1 can bind glucocorticoid receptors as a functional ligand, thereby exhibiting glucocorticoid effects [8]. Du et al. confirmed that Rg1 exerts dexamethasone-like anti-inflammatory effects on osteoblasts, not promoting hyperglycemia and osteoporosis, indicating less severe side-effects than glucocorticoids [8].

## 5. Conclusions

In conclusion, Ginsenoside Rg1 inhibited IL-1β-induced human chondrocyte gene and protein expressions of MMP-13, COX-2 and PGE2, and prevented type II collagen and aggrecan degradation. Administration of Ginsenoside Rg1 to OA rats attenuated cartilage degeneration, and reduced type II collagen loss and MMP-13 levels. These findings suggest that Rg1 has potential clinical benefits for OA treatment.
